# Phase Structure and Electrical Properties of Sm-Doped BiFe_0.98_Mn_0.02_O_3_ Thin Films

**DOI:** 10.3390/nano12010108

**Published:** 2021-12-30

**Authors:** Yangyang Wang, Zhaoyang Li, Zhibiao Ma, Lingxu Wang, Xiaodong Guo, Yan Liu, Bingdong Yao, Fengqing Zhang, Luyi Zhu

**Affiliations:** 1 School of Materials Science and Engineering, Shandong Jianzhu University, Jinan 250101, China; wyyedu0207@163.com (Y.W.); mzbsdjzu@163.com (Z.M.); wanglingxu@163.com (L.W.); yshy1589@163.com (Y.L.); bdyao1026@163.com (B.Y.); 2 State Key Laboratory of Crystal Materials, Institute of Crystal Materials, Shandong University, Jinan 250100, China; lzy08201226@163.com; 3 School of Data and Computer Science, Shandong Women’s University, Jinan 250300, China; sjgxd246@163.com

**Keywords:** BSFM, phase transition, aging, electrical properties

## Abstract

Bi_1__−x_Sm_x_Fe_0.__98_Mn_0__.02_O_3_ (x = 0, 0.02, 0.04, 0.06; named BSFMx) (BSFM) films were prepared by the sol-gel method on indium tin oxide (ITO)/glass substrate. The effects of different Sm content on the crystal structure, phase composition, oxygen vacancy content, ferroelectric property, dielectric property, leakage property, leakage mechanism, and aging property of the BSFM films were systematically analyzed. X-ray diffraction (XRD) and Raman spectral analyses revealed that the sample had both R3c and Pnma phases. Through additional XRD fitting of the films, the content of the two phases of the sample was analyzed in detail, and it was found that the Pnma phase in the BSFMx = 0 film had the lowest abundance. X-ray photoelectron spectroscopy (XPS) analysis showed that the BSFMx = 0.04 film had the lowest oxygen vacancy content, which was conducive to a decrease in leakage current density and an improvement in dielectric properties. The diffraction peak of (110) exhibited the maximum intensity when the doping amount was 4 mol%, and the minimum leakage current density and a large remanent polarization intensity were also observed at room temperature (2Pr = 91.859 μC/cm^2^). By doping Sm at an appropriate amount, the leakage property of the BSFM films was reduced, the dielectric property was improved, and the aging process was delayed. The performance changes in the BSFM films were further explained from different perspectives, such as phase composition and oxygen vacancy content.

## 1. Introduction

Only a small fraction of all magnetically polarized and electrically polarized materials are ferromagnetic or ferroelectric, and even fewer, namely multiferroic materials, have both properties [1,2]. In addition, the coupling between different properties of multiferroic materials will produce new properties, such as magnetoelectric effects. These materials have great development potential in the miniaturization and multi-functionalization of devices, as well as in a wide range of applications in the fields of magnetoelectric memory [3,4], sensors [5], and drivers [6]. Multiferroic materials are some of the most valuable multifunctional materials, and they have good application prospects in the field of multiferroic devices.

Multiferroic materials include single-phase materials and composite materials. However, few single-phase multiferroic materials have been discovered at present, and their Curie temperatures are usually low. Owing to its high Curie temperature (Tc = 1103 K) and Neal temperature (T_N_ = 647 K), single-phase BiFeO_3_ (BFO) exhibits ferroelectric and G-type antiferromagnetism at room temperature [7,8]. Thus, it has attracted extensive attention from materials scholars and has become a hot topic for in-depth exploration of multiferroic materials [9,10,11,12].

In BFO, Bi ions are volatile at high temperature. To balance the charge, the valence of Fe ions may change from +3 to +2 [13]:

(1) $${\mathrm{Bi}}_{\mathrm{Bi}}^{x}+\frac{3}{2}O_{2}\Leftrightarrow {\mathrm{Bi}}_{2}O_{3}↑+V_{\mathrm{Bi}}^{\prime \prime \prime }+3h^{•},$$

(2) $$2{\mathrm{Fe}}^{3+}+O_{O}^{x}\Leftrightarrow 2\left({\mathrm{Fe}}_{{\mathrm{Fe}}^{3+}}^{2+}\right)\prime +V_{O}^{••}+\frac{1}{2}O_{2}.\;$$

As a result, a large number of oxygen vacancies or other defects often exist in the prepared BiFeO_3_ samples, which increases the leakage current density of BiFeO_3_ materials and adversely affects its performance [14]. There are many ways to improve the properties of BiFeO_3_ materials, including element doping, solid solution, formation of a heterostructure, and control of film orientation [15,16,17,18]. Among them, many researchers adopt the element doping method to improve the performance of BiFeO_3_ materials [19,20,21,22]. Yun et al. prepared single-phase multiferroic BiFeO_3_ and Ho-doped BiFeO_3_ films [23]. The ferroelectric property was enhanced, and the leakage current decreased significantly. The ferroelectric property reached 20.69 μC/cm^2^ and the leakage current density was 2.89 × 10^−9^ A/cm^2^, and these effects were attributed to the transformation from a rhombohedral structure to a coexisting cubic and orthosymmetric structure after Ho doping. Moreover, the fatigue properties of the films doped with Ho also improved, as evidenced by a 0.4% reduction in the value of the switchable polarization. Liu et al. grew a Bi_1__−x_Eu_x_FeO_3_ (BEFOx, x = 0, 0.03, 0.05, 0.07, 0.1) thin film on LaNiO_3_-coated Si substrate by the pulse laser deposition method. As the doping amount increased, the position of the A_1_-1 mode of the films shifted to a higher wave number in the Raman spectrum [24]. With the increase in Eu, the refractive index of the film increased, and the extinction coefficient and band gap width decreased. Yang et al. prepared a BiFe_1__−x_Zn_x_O_3_ (BFZO) film (x = 0%, 1%, 2%, 3%) and found that when x = 2%, the film reached the maximum remanent polarization intensity and the minimum correction field [25]. At the same time, under a low electric field, Zn doping can significantly reduce the leakage current of BFO films. In addition, the leakage mechanism changes from Ohmic conduction under a low electric field to F-N tunneling under a high electric field. Zhang et al. prepared high-quality BiFe_1__−2x_Zn_x_Ti_x_O_3_ (BFZTO, x = 0, 0.01, 0.02, 0.03, 0.04, and 0.05) films [26]. The authors found that the BFZTO film with x = 0.02 had uniform fine grains and high density, which can inhibit the transformation of Fe^3+^ to Fe^2+^ and, thus, greatly reduce the oxygen vacancy concentration. This film had the lowest leakage current density and the highest remanent polarization intensity. By comparing P–E hysteresis loops in different areas of BiFe_0.96_Zn_0.02_Ti_0.02_O_3_ thin films, the films have high uniformity and stable properties. Concurrently, Zn and Ti co-doping also increased the dielectric permittivity from 24.9 to 35.3 and remnant magnetization from 0.05 to 0.80 emu/cm^3^ of BFZTO films. Liu et al. prepared Bi_0.9_Er_0.1_Fe_1__−x_Mn_x_O_3_ (BEFM_x_O, x = 0.00–0.03) thin films by the sol-gel method [27]. By co-doping Er and Mn, the coexistence of two phases (space groups are R3c:H and R3m:R) and the reduction of oxygen vacancy and Fe^2+^ concentration in BEFM_x_O were realized. Among all the samples, the BEFM_0.02_O film had the lowest oxygen vacancy concentration, the maximum remanent polarization value, and the maximum switching current. It also exhibited excellent ferroelectric stability, which means its low concentration of oxygen vacancies had less influence on the ferroelectric domains.

Kan et al. found that doping with Sm affected the phase structure of BFO samples [28,29]. Xue et al. prepared BFO films with different Sm content by the sol-gel method and found that the rhombohedral phase to pseudo-tetragonal phase transition occurs gradually with the increase in Sm [30]. Although there are many studies on the influence of element doping on BiFeO_3_ properties, there are few on the influence of Sm doping on the content change in the BiFeO_3_ thin film phase structure and thus on ferroelectric properties. In addition, the literature review revealed that for Sm doping, when the doping content is less than 10 mol%, BFO has better properties than heavily doped [31,32]. It is necessary to further adjust the doping content. In this experiment, the doping amounts of Sm were 2 mol%, 4 mol%, and 6 mol%, in order to understand the influence of Sm doping on BSFM films. The performance changes were analyzed in detail from the aspects of oxygen vacancy content, grain size, relative content of the R3c phase and the Pnma phase. Additionally, the effects of different Sm content on the ferroelectric, dielectric, leakage, and aging properties of the thin film samples were systematically studied.

## 2. Materials and Methods

Bi_1-x_Sm_x_Fe_0.98_Mn_0.02_O_3_ thin films (x = 0, 0.02, 0.04, 0.06) were prepared by the sol-gel method on ITO/glass substrate. Fe(NO_3_)_3·_9H_2_O (purity of 98.5%), Bi(NO_3_)_3_·5H_2_O (purity of 98.5%), Sm(NO_3_)_3_·6H_2_O (purity of 98.5%), and MnC_4_H_6_O_4_·4H_2_O (purity of 98.5%) were taken as solutes, according to stoichiometric ratio. Bi excess of 5% compensated for bismuth volatilization during high-temperature annealing. The solutes were successively added to a solvent mixture of CH_3_COOH and C_2_H_6_O, with a volume ratio of 3:1, and stirred at room temperature at a uniform speed until completely dissolved. Then, C_5_H_8_O_2_ was added to the solution as a chelating agent and stirred at room temperature for 12 h at a constant speed to obtain a red-brown and transparent precursor solution. Finally, the stable precursor solution of 0.3 mol/L was obtained by allowing the precursor solution to rest for 24 h. Then, the BSFM precursor solution was rotated onto ITO/glass substrate, and the film was coated at 3500 r/min. The wet film was dried on an electric heating plate at 250 °C to remove excess organic solvents and water. It was then placed in an annealing furnace and annealed at 550 °C. The coating was repeated, and the film was dried and annealed 10 times to obtain the desired samples.

Before testing the electrical properties of the sample, Au was sputtered on the surface of the sample to achieve the effect of conduction. We used a small-ion sputtering instrument (JS-1600, Beijing Hetong Venture Technology Co., Ltd., Beijing, China) to complete this process. The samples were characterized by an X-ray diffractometer (D8-Advance, Drudy, Germany) recorded in the 2-theta range of 20–60° with a step of 0.02°, and by a microconfocal Raman spectrometer (HR800, LabRAM, Horiba Co., Palaiseau, France) to measure in the shift range of 50–650 cm^−1^. The Fe and O elements in the samples were analyzed by a Wscabb X-ray photoelectron spectrometer. A dielectric tester (TH2828, Xintonghui Electronics Co., Ltd., Suzhou, China) was used to test the dielectric properties of the samples in the range of 1 kHz–1 MHz with an oscillation voltage of 1 V. The ferroelectric properties at 1 kHz and leakage properties of the samples were measured via a multiferroic tester (Radiant Co., Albuquerque, NM, USA). 

## 3. Results and Discussion

**Figure 1:**
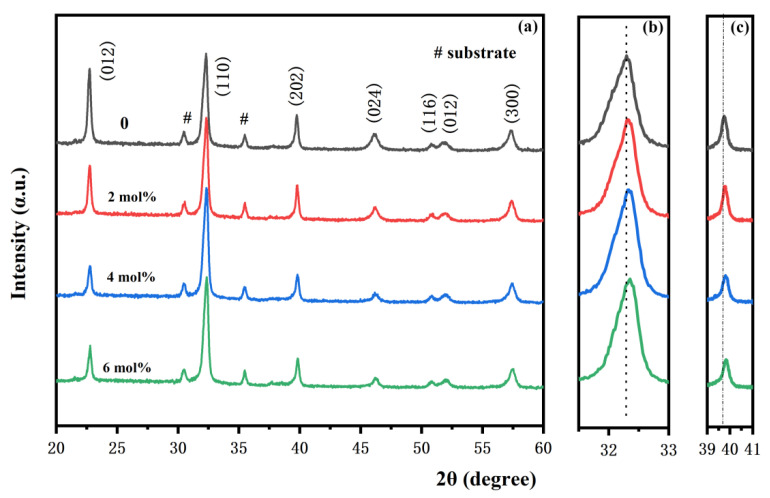
(**a**) XRD patterns, (**b**) magnified patterns around 30.5–33°, and (**c**) magnified patterns around 39–41° of the BSFM films with different Sm content.

Figure 1a shows the XRD patterns of Bi_1-x_Sm_x_Fe_0.98_Mn_0.02_O_3_ (x = 0, 0.02, 0.04, 0.06) films deposited on ITO/glass substrate. Figure 1b,c show the local magnified diffraction peaks of the (110) and (202) crystal planes, respectively. From the XRD pattern, the generated sample had a polycrystalline perovskite structure, and the films had good crystallinity, which matches well with the JCPDS (No. 86-1518) PDF standard card. However, the peak-splitting phenomenon of the rhombohedral structure was not observed in the BFSM thin films. At 2θ = 32°, the BSFM thin film samples do not show (110)/(104) peak splitting, but preferentially grow along (110). This may be due to the structural phase transition in the BSFM films. Figure 1b,c show that with the increase in Sm doping amount, the diffraction peak of (110) and the diffraction peak of (202) gradually shift to a large angle, which may be because the radii of doped Sm^3+^ (0.96 Å) and Mn^2+^ (0.67Å) are smaller than that of Bi^3+^ (1.03 Å) and Fe^3+^ (0.64 Å) [33,34]. Lattice distortion occurred during doping, and the crystal plane spacing decreased. In addition, with the increase in Sm content, the relative strengths I = I_(110)_/I_(012)_ of BSFMx = 0.02, BSFMx = 0.04, BSFMx = 0.06 were 2.03, 3.70, 3.02, respectively. When the content was 4 mol%, the relative intensity reached the maximum value, and the intensity of diffraction peak (202) did not change significantly with doping amount.

**Figure 2:**
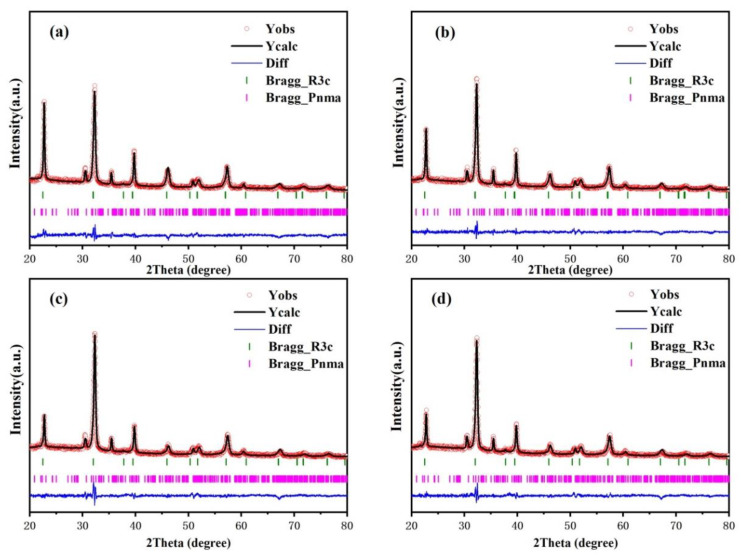
Rietveld refined XRD patterns of the BSFM films with different Sm content: (**a**) x = 0 mol%; (**b**) x = 2 mol%; (**c**) x = 4 mol%; (**d**) x = 6 mol%.

The above data indicate that the crystal had structural distortion. To further study the changes in crystal structure, Rietveld refinement was conducted on all samples, and the results are shown in Figure 2a–d. All samples were used for two-phase refinement using R3c and Pnma cards from the International Crystallography database. The phase content and structural parameters of the samples are shown in Table 1. According to the experimental data, all samples have R3c and Pnma space groups. With the increase in Sm content, the content of R3c phase in the samples was 70.98%, 68.39%, 69.06%, and 67.06%. The addition of Sm reduced the content of R3c phase in the BSFM films. The lone electron pair 6S^2^ of Bi^3+^ in BiFeO_3_ is chemically active, and it is conducive to ferroelectric distortion [20]. The substitution of rare earth element Sm for Bi may reduce the chemical activity of the lone electron pair and reduce the rhombohedral distortion of the crystal. In all Sm-doped samples, the content of the R3c phase in BSFMx = 0.04 was the highest, and the content of the non-polar orthorhombic phase Pnma was the lowest [35,36].

**Figure 3:**
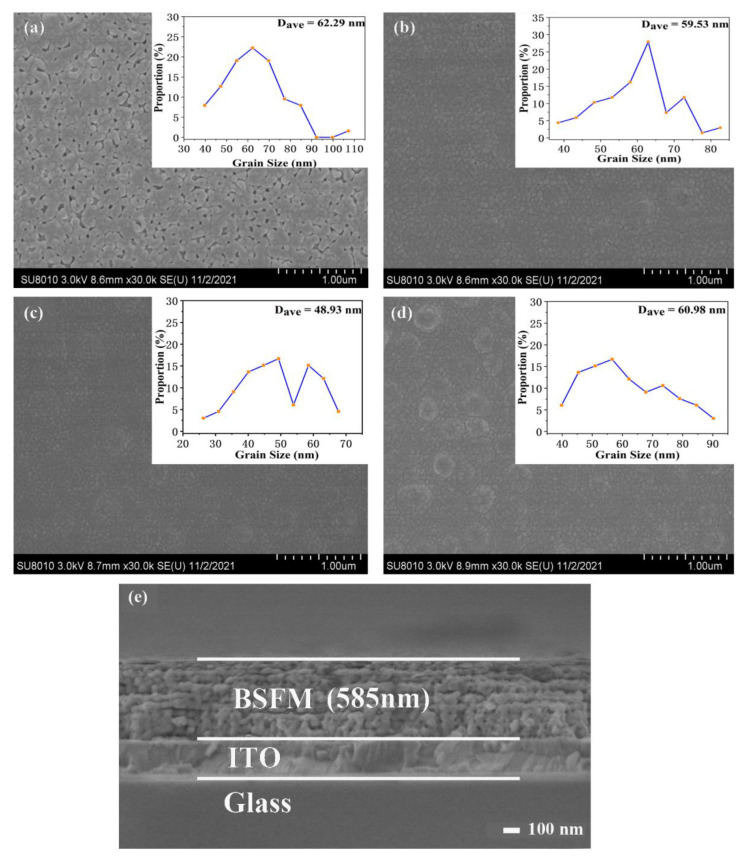
SEM images of the surface morphological features of the BSFM films: (**a**) x = 0 mol%; (**b**) x = 2 mol%; (**c**) x = 4 mol%; (**d**) x = 6 mol%, and (**e**) the cross-sectional image of the BSFMx = 0.06 film. The insets show the distribution of crystal grain size and the average grain diameter (D_ave_).

The surface differentiation features of the BSFMx (x = 0–0.06) films are shown in Figure 3a–d. The crystal grain size of the BSFMx (x = 0–0.06) films are shown in the inset. The figure shows that the grain distribution on the surface of the film without Sm doping is not uniform, and that there are many voids, which may be the reason for the volatilization of the organic solution. Furthermore, the surfaces of the Sm-doped films were uniform, compact, and well combined with the substrate, indicating that the annealing mechanism was very suitable for the growth of the BSFM films on the ITO substrate. The average grain sizes of the BSFMx (x = 0–0.06) films were 62.29, 59.53, 48.93, and 60.98 nm, indicating that Sm doping can reduce grain size. Among them, the BSFMx = 0.04 film had the smallest grain size, suggesting that appropriate Sm doping accelerated the nucleation rate and decreased the grain size [37]. The cross-sectional image of the BSFMx = 0.06 film is shown in Figure 3e. As can be seen from the figure, the film has a clear interface with the substrate, and the cross-section thickness of the film is 585 nm.

**Figure 4:**
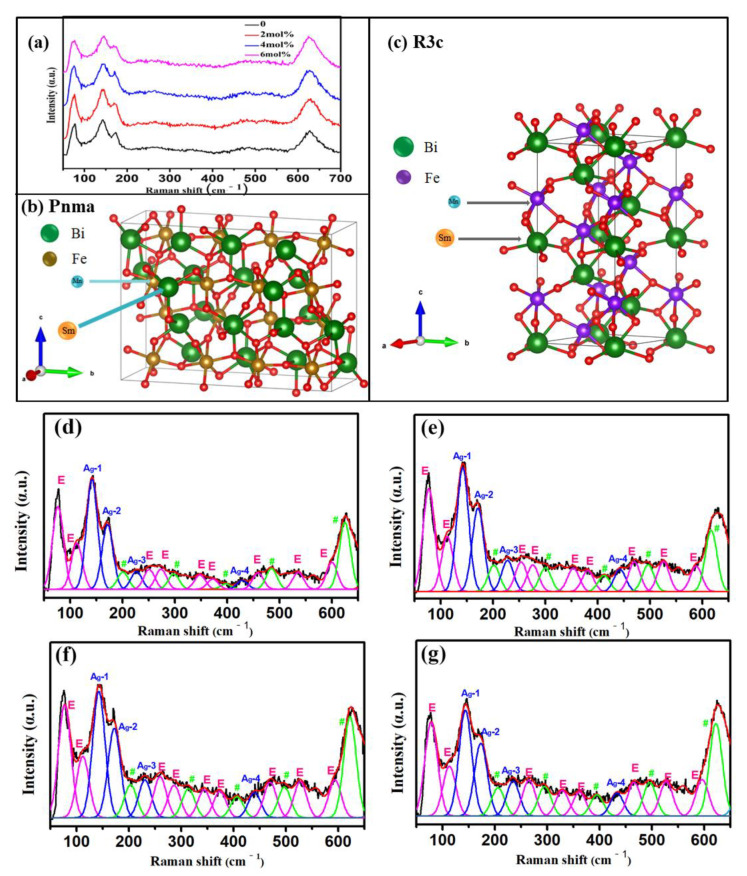
(**a**) Raman spectra of the BSFM films with different Sm content and crystal structure of the BSFM films: (**b**) Pnma; (**c**) R3c, Raman fitting spectra of the BSFM films with different Sm content: (**d**) x = 0 mol%; (**e**) x = 2 mol%; (**f**) x = 4 mol%; (**g**) x = 6 mol%. # refers to the Raman frequencies of the Pnma structure.

To further analyze the structure of Sm-doped BSFM thin films, Raman spectroscopy was used. Figure 4a shows the Raman spectra of BSFMx (x = 0−0.06) films with different Sm content in the wave number range of 50 cm^−1^–650 cm^−1^. The data for the crystal structure of the BSFM films (Pnma+R3c) according to XRD-refined parameters and developed by FullProf software are shown in Figure 4b,c. Figure 4d–g are the Raman spectrum fitting diagrams of each sample. Group theory analysis shows that the vibration modes of the BFO film with the R3c space group with a rhombohedral perovskite structure are Γ_Raman,R3c_ = 4A_1_ + 9E [38]. Four A_1_ and nine E vibration modes analyzed by group theory were observed in the Raman spectra of the BSFM films. The Raman vibration modes extracted from the Raman fitting are listed in Table 2. From the Raman fitting diagram, the strength of mode A in the BSFM films significantly increased, which may be related to the change in Bi-O bonds caused by Sm^3+^ replacing Bi^3+^. Owing to the Jahn–Teller distortion effect [39], the strength of modes E-8 and E-9 improved. This is because changes in Bi-O bonds lead to the distortion of the ferrite octahedron, which further changes the Fe-O bond. In addition, when the Sm element was added, the A_1_-1 vibration mode shifted to a higher wave number (from 141.76 cm^−1^ to 143.31 cm^−1^), because the frequency of the Raman vibration mode is related to the functions of ion mass and force [22]. Sm^3+^ (150.4 g) replaces Bi^3+^ (209.0 g), which leads to a blue shift in the A_1_-1 mode. These results show that Sm^3+^ doping causes lattice distortion, which is consistent with XRD analysis. In addition, all samples had vibration patterns near 200, 300, 400, 490, and 620 cm^−^^1^, which were consistent with the Raman frequencies of the Pnma structure [40]. In Figure 4c–f, the vibration patterns are indicated by #. The results show that in addition to the R3c phase, the Pnma phase also existed in all samples, which was consistent with the XRD refinement results.

**Figure 5:**
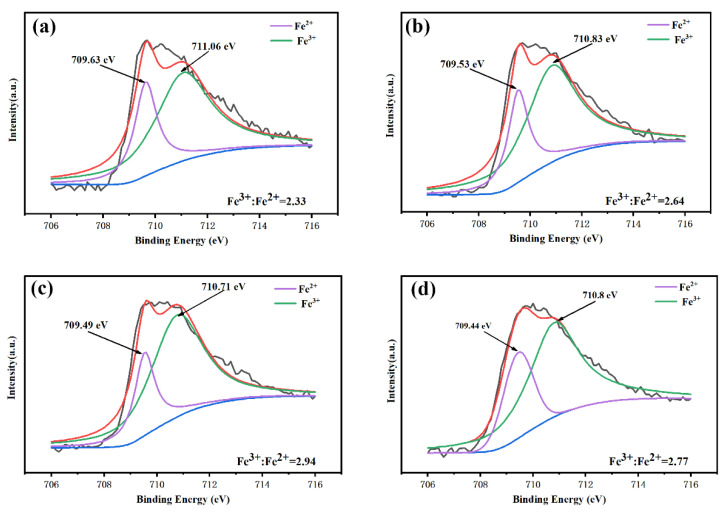
Fe2p_3/2_ orbital XPS fitting diagram of the BSFM films with different Sm content: (**a**) x = 0 mol%; (**b**) x = 2 mol%; (**c**) x = 4 mol%; (**d**) x = 6 mol%. The black lines refer to the original curve, the red lines refer to the fitting curve, and the blue lines refer to the background line.

According to the defect equation (Formula (2)) [13], the Fe^2+^ content can affect the oxygen vacancy content. Thus, the Fe2p_2/3_ orbit of the BSFM films with different Sm doping amount was fitted, as shown in Figure 5a–d. The Fe2p_2/3_ peaks were fitted into two peaks corresponding to Fe^2+^ and Fe^3+^. From further calculations, the Fe^3+^:Fe^2+^ ratios of the BSFMx (x = 0–0.06) films were 2.33, 2.64, 2.94, and 2.77. The content of Fe^2+^ in the BSFMx = 0.04 film was the lowest, indicating that an appropriate amount of Sm doping can effectively inhibit the variation of Fe element. Generally, the content of Fe^2+^ will affect the generation of oxygen vacancies. The lower the content of Fe^2+^, the fewer oxygen vacancies will be generated, and the fewer defects will exist in the samples.

**Figure 6:**
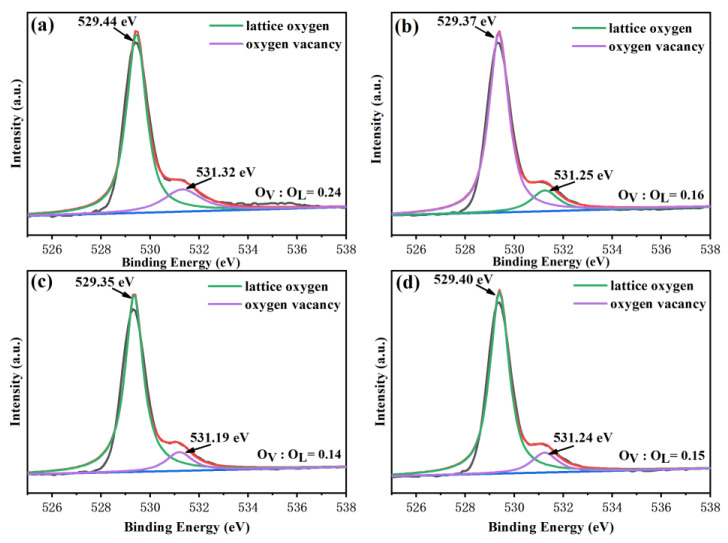
O1s orbital XPS fitting diagram of the BSFM films with different Sm content: (**a**) x = 0 mol%; (**b**) x = 2 mol%; (**c**) x = 4 mol%; (**d**) x = 6 mol%. The black lines refer to the original curve, the red lines refer to the fitting curve, and the blue lines refer to the background line.

To further study the oxygen vacancy content in the BSFM films, the O1s orbital of the films was fitted, as shown in Figure 6a–d. In the figure, O1s is fitted into two peaks, which are lattice oxygen with binding energy of 529 eV (from metal) and oxygen vacancy of 531 eV (from defect) [41,42]. To calculate the oxygen vacancy ratio of the thin films, the two peaks were integrated to calculate the area, and the oxygen vacancy ratios of the four groups of samples were 0.24, 0.16, 0.14, and 0.15. According to the calculation results, the oxygen vacancy content and the Fe2p_3/2_ orbital Fe^2+^ content of the BSFMx = 0.04 film were the lowest. This further confirms the view that inhibiting the transformation of Fe^3+^ to Fe^2+^ reduces the oxygen vacancy content of the sample.

**Figure 7:**
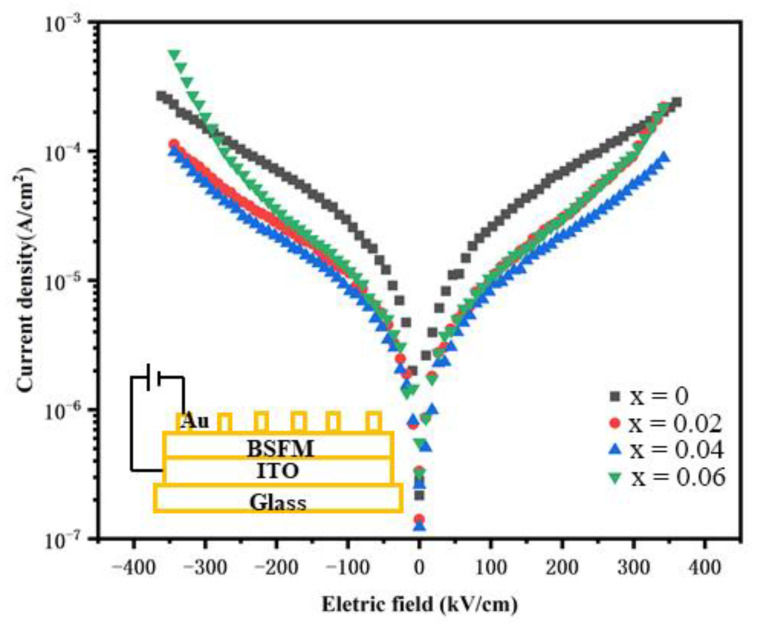
Leakage current density curves of the BSFM films with different Sm content.

Figure 7 shows the leakage current density curve (J–E) of the BSFM film with different Sm content. The test electric field was 350 kV/cm. The asymmetry of leakage current of positive and negative electric fields can be attributed to the different work functions of the upper and lower electrodes. With an increasing doping amount, the leakage current density decreased first and then increased. Under the same electric field, when the doping amount was 4 mol%, the leakage current density reached the minimum value. Under a 350 kV/cm electric field, the leakage current density of the BSFMx = 0.04 film reached 8.84 × 10^−5^ A/cm^2^. The leakage current density was about 0.5 orders of magnitude lower than that of the pure BiFe_0.98_Mn_0.02_O_3_ film. Under normal circumstances, the leakage current of the sample was affected by oxygen vacancy content, which is mainly related to the high-temperature volatilization of Bi^3+^ and the variation in Fe^3+^. It can be seen from XPS that the Fe^2+^ content and the oxygen vacancy content in the BSFMx = 0.04 film was the lowest, resulting in the minimum leakage current density. In addition, grain boundaries hinder electron migration and reduce leakage current density. According to the SEM results, the BSFMx = 0.04 film has the smallest grain size, indicating that it has more grain boundaries and greater resistance to electron migration [26,42].

**Figure 8:**
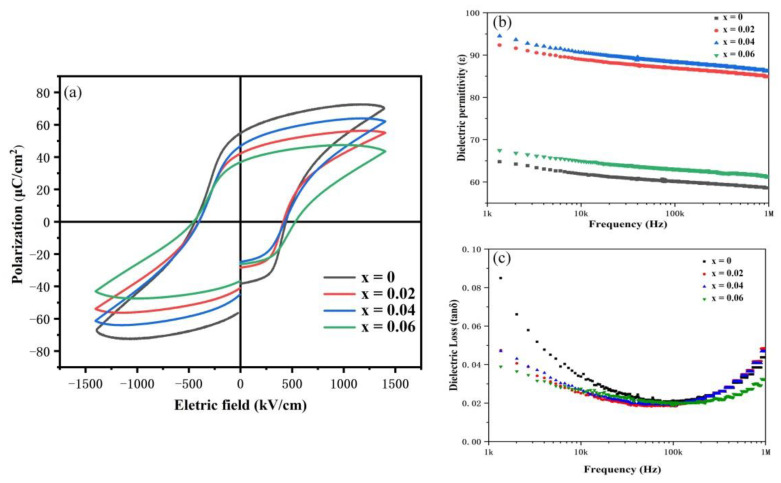
(**a**) P−E hysteresis loops, (**b**) dielectric permittivity, and (**c**) dielectric loss of the BSFM films with different Sm content.

Figure 8a shows the hysteresis loops of the BSFM films with different Sm content with an electric field of 1410 kV/cm and frequency of 1 kHz. The remanent polarization intensities of BSFMx (x = 0−0.06) under the test electric field were 111.23, 83.30, 91.86, and 73.59 μC/cm^2^. The coercive field Ec was 835.22, 819.28, 830.95, and 987.55 kV/cm, respectively. When the content was less than or equal to 4 mol%, the change in the coercive field was small. The defects inhibit ferroelectric domain flipping. In all the Sm-doped samples, the remanent polarization value increased first and then decreased with increasing doping content. When the doping content was 4 mol%, the remanent polarization value was the largest, and the ferroelectric property was the best, which was consistent with the XRD analysis results. The possible reasons are as follows: (1) The leakage current density of BSFMx = 0.04 has a minimum value compared with other samples. A smaller leakage current density can improve the voltage resistance of the sample and facilitate electric domain inversion [28]. (2) According to XPS experimental data, the BSFMx = 0.04 sample had the lowest oxygen vacancy content, and the reduction of oxygen vacancy will improve its ferroelectric performance. (3) Owing to its centrosymmetric characteristics, the Pnma phase shows paramagnetic properties without ferroelectric properties, while the R3c phase mainly affects the ferroelectric property of samples [43,44,45].

Figure 8b,c show that the dielectric permittivity (ε) and dielectric loss (tanδ) of the BSFM films with different Sm contents vary with test frequency at room temperature in the range of 1 kHz–1 MHz. From the figure, the dielectric permittivity of the sample has little dependence on frequency, indicating that the sample has high intermediate frequency stability. The results show that the dielectric permittivity values of BSFMx (x = 0−0.06) at 10 kHz were 62, 89, 91, and 65. At the same frequency, the dielectric losses of BSFMx (x = 0−0.06) were 0.034, 0.025, 0.026, and 0.027. In the low frequency range (<40 kHz), Sm doping reduced the dielectric loss of the BSFM films. At the same time, Sm doping increased the dielectric permittivity of the BSFM films, and the maximum dielectric permittivity was obtained when the doping amount was 4 mol%. The existence of oxygen vacancies will distort the free volume used to replace Fe^3+^ in the Fe-O octahedron, which reduces the dielectric polarization and leads to a smaller dielectric permittivity [46]. As a result, the BSFMx = 0.04 film with the lowest oxygen vacancy content has the maximum dielectric permittivity.

**Figure 9:**
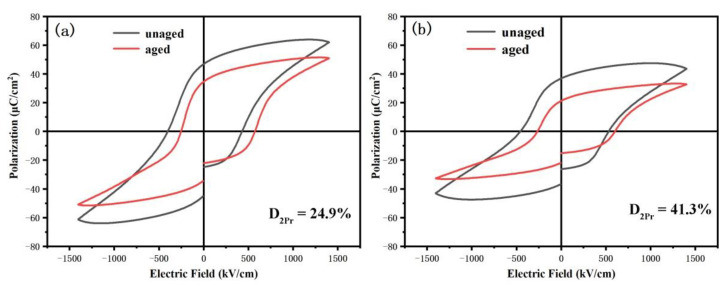
Comparison of hysteresis loops of the BSFM films with different Sm content measured after an interval of 110 days: (**a**) x = 4 mol%; (**b**) x = 6 mol%.

BSFMx = 0.04 and BSFMx = 0.06 films were selected as representatives to explain the aging behavior of samples. Figure 9 shows the electrical hysteresis loop diagram of the BSFM films aged at room temperature for 110 d. The remanent polarization strength (2Pr) of the BSFMx = 0.04 and BSFMx = 0.06 films after aging treatment decreased by 24.9% and 41.3%, respectively, while the intensity of the coercive electric field (2Ec) decreased by 0.6% and 12.2%, respectively. This indicates that the two samples have different degrees of aging. Among them, the aging degree of the BSFMx = 0.04 film was smaller. Ren et al. proposed the symmetric short-range order principle of point defects and inferred that the aging effect could be triggered by ion doping [47,48,49,50]. Moreover, the migration of oxygen vacancies led to the formation of complex defect dipoles in the sample. The symmetrical short-range order of oxygen vacancies created conditions suitable for reversible domain switching. In addition, the internal electric field formed during the orderly arrangement of the defective dipoles will increase the offset of the coercive field, which directly explains the asymmetry of the coercive field strength of the sample shown in Figure 9. The domains within ferroelectrics can be switched under an applied electric field, exhibiting macroscopic polarization. However, when the applied electric field is removed, the domain structure gradually shifts to a random state, resulting in the degradation of properties over time, namely, sample aging. The movement of oxygen vacancies in the sample to the domain walls creates pin centers that provide resistance to the movement of the domain walls and reduce the mobility of the domain walls. This, in turn, affects the ferroelectric properties associated with the movement of the domain walls. According to the XPS data, compared with the BSFMx = 0.06 film, the oxygen vacancy content in the BSFMx = 0.04 film was less, which means that the defect dipole content of the sample was smaller. Thus, the resistance of the domain wall to movement was smaller, leading to weak aging effects and better retention of film performance. In addition, it can be seen from SEM images that the BSFMx = 0.04 film had a smaller grain size than the BSFMx = 0.06 film. For small-grain crystals, due to the small difference between lattice symmetry and defect symmetry, the thermodynamic force driving the symmetry matching between them is weak, and this hinders the kinetic migration of oxygen vacancies and affects reversible domain switching [47,51]. Therefore, the BSFMx = 0.04 film with small grains aged slowly.

## 4. Conclusions

In conclusion, Sm-doped BSFM films were prepared on ITO/glass substrates by the sol-gel method. The effects of different Sm content on the leakage current density, dielectric properties, and aging properties of the BSFM films were systematically studied. Detailed explanations were made in terms of phase transition and oxygen vacancy. XRD and Raman analyses show that the samples all contained R3c and Pnma phases, and the samples with different Sm content had different phase composition. Sm doping led to lattice structure distortion and decreases in the crystal plane spacing. XPS analysis showed that the BSFMx = 0.04 thin film sample had the lowest oxygen vacancy content, indicating that an appropriate amount of Sm doping can effectively inhibit the valence of the Fe element. The decrease in oxygen vacancy increased the dielectric permittivity and the leakage current density. The minimum leakage current density of the BSFMx = 0.04 film sample was 8.84 × 10^−5^ A/cm^2^ in a 350 kV/cm electric field. The remanent polarization intensity of the BSFMx = 0.04 film was 91.86 μC/cm^2^, owing to the formation of avnonpolar orthorhombic Pnma phase during the doping process. At the same time, the aging process of BSFMx = 0.04 sample was slow, and the performance of the sample was better preserved.

## Figures and Tables

**Table 1:** Rietveld refinement parameters of the BSFM films with different Sm content.

Sample	Space Group	Fraction (%)	Lattice Parameter							R_w_ (%)
			a (Å)	b (Å)	c (Å)	α (°)	β (°)	γ (°)	Volume	
BSFMx = 0	R3c	70.98	5.59	5.59	13.70	90	90	120	370.41	7.90
	Pnma	29.02	5.60	16.01	11.28	90	90	90	1010.60	
BSFMx = 0.02	R3c	68.39	5.58	5.58	13.70	90	90	120	369.56	6.83
	Pnma	31.61	5.62	15.98	11.26	90	90	90	1010.75	
BSFMx = 0.04	R3c	69.06	5.58	5.58	13.66	90	90	120	368.67	7.40
	Pnma	30.94	5.58	16.06	11.30	90	90	90	1012.96	
BSFMx = 0.06	R3c	67.06	5.58	5.58	13.67	90	90	120	368.65	7.17
	Pnma	32.94	5.56	16.06	11.28	90	90	90	1007.00	

**Table 2:** The Raman modes of the BSFM films with different Sm content.

Raman Modes (cm^−1^)	BSFMx = 0	BSFMx = 0.02	BSFMx = 0.04	BSFMx = 0.06
E	76.67	76.99	77.392	78.053
E	112.61	112.45	112.03	112.80
A_1_-1	141.76	141.88	142.64	143.31
A_1_-2	171.43	171.05	172.11	172.89
pnma	200.74	201.38	203.34	205.78
A_1_-3	226.61	227.47	230.80	234.49
E	251.23	253.53	259.69	264.92
E	273.72	275.8	286.47	---
pnma	298.05	299.78	313.95	294.87
E	348.13	353.32	343.83	---
E	372.68	380.91	374.02	361.13
pnma	397.33	411.95	406.72	394.90
A_1_-4	428.90	442.21	440.26	432.93
E	459.09	469.92	470.06	465.64
pnma	484.76	496.11	497.29	495.72
E	535.62	525.24	527.48	527.32
E	600.15	587.81	593.70	595.79
pnma	624.89	616.55	622.52	621.96

## Data Availability

Not applicable.

## References

[1] Martin L.W., Ramesh R. (2012). Overview No. 151 Multiferroic and magnetoelectric heterostructures. Acta Mater..

[2] Eerenstein W., Mathur N.D., Scott J.F. (2006). Multiferroic and magnetoelectric materials. Nature.

[3] Bibes M., Barthelemy A. (2008). Multiferroics: Towards a magnetoelectric memory. Nat. Mater..

[4] Yue Z.W., Tan G.Q., Yang W., Ren H.J., Xiao A. (2016). Enhanced multiferroic properties in Pr-doped BiFe_0.97_Mn_0.03_O_3_ films. Ceram. Int..

[5] Sreenivasulu G., Laletin U., Petrov V.M., Petrov V.V., Srinivasan G. (2012). A permendur-piezoelectric multiferroic composite for low-noise ultrasensitive magnetic field sensors. Appl. Phys. Lett..

[6] Hasegawa M., Asano T., Hashimoto K., Lee G.C., Park Y.C., Okazaki T., Furuya Y. (2006). Fabrication of multiferroic composite actuator material by combining superelastic TiNi filler and a magnetostrictive Ni matrix. Smar. Mate. Stru..

[7] Huang A., Handoko A.D., Goh G.K., Pallathadka P.K., Shannigrahi S. (2010). Hydrothermal synthesis of (001) epitaxial BiFeO_3_ films on SrTiO_3_ substrate. CrystEngComm.

[8] Lebeugle D., Colson D., Forget A., Viret M., Bonville P., Marucco J.F., Fusil S. (2007). Room-temperature coexistence of large electric polarization and magnetic order in BiFeO_3_ single crystals. Phys. Rev. B.

[9] Prashanthi K., Shaibani P.M., Sohrabi A., Natarajan T.S., Thundat T. (2012). Nanoscale magnetoelectric coupling in multiferroic BiFeO_3_ nanowires. Phys. Status Solidi RRL.

[10] Shami M.Y., Awan M.S., Anis-ur-Rehman M. (2012). The effect of heat treatment on structural and multiferroic properties of phase-pure BiFeO_3_. J. Electron. Mater..

[11] Singh S.K. (2013). Structural and electrical properties of Sm-substituted BiFeO_3_ thin films prepared by chemical solution deposition. Thin Solid Film..

[12] Zhou J., Trassin M., He Q., Tamura N., Kunz M., Cheng C., Wu J. (2012). Directed assembly of nano-scale phase variants in highly strained BiFeO_3_ thin films. J. Appl. Phys..

[13] Reetu A., Sanghi S. (2011). Rietveld analysis, dielectric and magnetic properties of Sr and Ti codoped BiFeO_3_ multiferroic. J. Appl. Phys..

[14] Qi X., Dho J., Tomov R., Blamire M.G., Blamire J.L. (2005). Greatly reduced leakage current and conduction mechanism in aliovalent-ion-doped BiFeO_3_ Macmanus-driscoll. Appl. Phys. Lett..

[15] Gumiel C., Jardiel T., Calatayud D.G., Vranken T., Van Bael M.K., Hardy A., Peiteado M. (2020). Nanostructure stabilization by low-temperature dopant pinning in multiferroic BiFeO_3_-based thin films produced by aqueous chemical solution deposition. J. Mater. Chem. C.

[16] Madolappa S., Kundu S., Bhimireddi R., Varma K.B. (2016). Improved electrical characteristics of Pr-doped BiFeO_3_ ceramics prepared by sol-gel route. Mater. Res. Express.

[17] Shimada T., Arisue K., Kitamura T. (2012). Strain-induced phase transitions in multiferroic BiFeO_3_ (110) epitaxial film. Phys. Lett. A.

[18] Yang J.C., He Q., Suresha S.J., Kuo C.Y., Peng C.Y., Haislmaier R.C., Chu Y.H. (2012). Orthorhombic BiFeO_3_. Phys. Rev. Lett..

[19] Chai Z., Tan G., Yue Z., Yang W., Guo M., Ren H., Lv L. (2018). Ferroelectric properties of BiFeO_3_ thin films by Sr/Gd/Mn/Co multi-doping. J. Alloys Compd..

[20] Wen X.L., Chen Z., Liu E.H., Lin X., Chen C. (2016). Effect of Ba and Mn doping on microstructure and multiferroic properties of BiFeO_3_ ceramics. J. Alloys Compd..

[21] Karpinsky D.V., Pakalniškis A., Niaura G., Zhaludkevich D.V., Zhaludkevich A.L., Latushka S.I., Kareiva A. (2021). Evolution of the crystal structure and magnetic properties of Sm-doped BiFeO_3_ ceramics across the phase boundary region. Ceram. Int..

[22] Sati P.C., Kumar M., Chhoker S. (2015). Phase evolution, magnetic, optical and dielectric properties of Zr-substituted Bi_0.9_Gd_0.1_FeO_3_ multiferroics. J. Am. Ceram. Soc..

[23] Yun Q., Bai Y.L., Chen J., Gao W., Bai A., Zhao S. (2014). Improved ferroelectric and fatigue properties in Ho doped BiFeO_3_ thin films. Mater. Lett..

[24] Liu J., Deng H.M., Zhai X., Lin T., Meng X., Zhang Y., Chu J. (2014). Influence of Zn doping on structural, optical and magnetic properties of BiFeO_3_ films fabricated by the sol-gel technique. Mater. Lett..

[25] Yang S.J., Zhang F.Q., Xie X., Sun H., Zhang L., Fan S. (2018). Enhanced leakage and ferroelectric properties of Zn-doped BiFeO_3_ thin films grown by sol-gel method. J. Alloys Compd..

[26] Zhang C.C., Dai J.Q., Liang X.L. (2021). Enhanced ferroelectric properties of (Zn, Ti) equivalent co-doped BiFeO_3_ films prepared via the sol-gel method. Ceram. Int..

[27] Liu Y., Tan G.Q., Ren X.X., Li J., Xue M., Ren H., Liu W. (2020). Electric field dependence of ferroelectric stability in BiFeO_3_ thin films co-doped with Er and Mn. Ceram. Int..

[28] Kan D., Pálová L., Anbusathaiah V., Cheng C.J., Fujino S., Nagarajan V., Takeuchi I. (2010). Universal behavior and electric-field-induced structural transition in rare-earth-substituted BiFeO_3_. Adv. Funct. Mater..

[29] Emery S.B., Cheng C.J., Kan D., Rueckert F.J., Alpay S.P., Nagarajan V., Wells B.O. (2010). Phase coexistence near a morphotropic phase boundary in Sm-doped BiFeO_3_ films. Appl. Phys. Lett..

[30] Xue X., Tan G.Q., Ren H.J. (2013). Structural, electric and multiferroic properties of Sm-doped BiFeO_3_ thin films prepared by the sol-gel process. Ceram Int..

[31] Tao H., Lv J., Zhang R., Xiang R., Wu J. (2017). Lead-free rare earth-modified BiFeO_3_ ceramics: Phase structure and electrical properties. Mater. Des..

[32] Zhang F., Zeng X., Bi D., Guo K., Yao Y., Lu S. (2018). Dielectric, ferroelectric, and magnetic properties of Sm-doped BiFeO_3_ ceramics prepared by a modified solid-state-reaction method. Materials.

[33] Liang X.L., Dai J.Q. (2021). Prominent ferroelectric properties in Mn-doped BiFeO_3_ spin-coated thin films. J. Alloys Compd..

[34] Zhou W., Deng H., Cao H., He J., Liu J., Yang P., Chu J. (2015). Effects of Sm and Mn co-doping on structural, optical and magnetic properties of BiFeO_3_ films prepared by a sol-gel technique. Mater. Lett..

[35] Goian V., Kamba S., Greicius S., Nuzhnyy D., Karimi S., Reaney I. (2011). Terahertz and infrared studies of antiferroelectric phase transition in multiferroic Bi_0. 85_Nd_0.15_FeO_3_. J. Appl. Phys..

[36] Gu Y., Zhou Y., Zhang W. (2021). Optical and magnetic properties of Sm-doped BiFeO_3_ nanoparticles around the morphotropic phase boundary region. AIP Adv..

[37] Li W., Hao J., Du J., Fu P., Sun W., Chen C., Chu R. (2020). Electrical properties and luminescence properties of 0.96(K_0.48_Na_0.52_)(Nb_0.95_Sb_0.05_)–0.04Bi_0.5_(Na_0.82_K_0.18_)_0.5_ZrO_3_-xSm lead-free ceramics. J. Adv. Ceram..

[38] Singh M.K., Jang H.M., Ryu S., Jo M.H. (2006). Polarized Raman scattering of multiferroic BiFeO_3_ epitaxial films with rhombohedral R3c symmetry. Appl. Phys. Lett..

[39] Wang Y., Nan C.W. (2008). Site modification in BiFeO_3_ thin films studied by Raman spectroscopy and piezoelectric force microscopy. J. Appl. Phys..

[40] Iliev M.N., Abrashev M.V., Lee H.G., Popov V.N., Sun Y.Y., Thomsen C., Chu C.W. (1998). Raman spectroscopy of orthorhombic perovskitelike YMnO_3_ and LaMnO_3_. Phys. Rev. B.

[41] Singh D., Tabari T., Ebadi M., Trochowski M., Yagci M.B., Macyk W. (2019). Efficient synthesis of BiFeO_3_ by the microwave-assisted sol-gel method: “A” site influence on the photoelectron chemical activity of perovskites. Appl. Surf. Sci..

[42] Wang J., Luo L., Han C., Yun R., Tang X., Zhu Y., Feng Z. (2019). The microstructure, electric, optical and photovoltaic properties of BiFeO_3_ thin films prepared by low temperature sol-gel method. Materials.

[43] Ma Z.B., Liu H.Y., Wang L.X., Zhang F.Q., Zhang F., Zhu L., Fan S. (2020). Phase transition and multiferroic properties of Zr-doped BiFeO_3_ thin films. J. Mater. Chem. C.

[44] Bai H., Li J., Hong Y., Zhou Z. (2020). Enhanced ferroelectricity and magnetism of quenched (1−x) BiFeO_3_-xBaTiO_3_ ceramics. J. Adv. Ceram..

[45] Hanani Z., Merselmiz S., Danine A., Stein N., Mezzane D., Amjoud M.B. (2020). Enhanced dielectric and electrocaloric properties in lead-free rod-like BCZT ceramics. J. Adv. Ceram..

[46] Lou Y.H., Song G.L., Chang F.G. (2010). Investigation on dependence of BiFeO_3_ dielectric property on oxygen content. Chin. Phys. B.

[47] Ren X., Otsuka K. (2000). Universal symmetry property of point defects in crystals. Phys. Rev. Lett..

[48] Ren X. (2004). Large electric-field-induced strain in ferroelectric crystals by point-defect-mediated reversible domain switching. Nat. Mater..

[49] Zhang L., Ren X. (2006). Aging behavior in single-domain Mn-doped BaTiO_3_ crystals: Implication for a unified microscopic explanation of ferroelectric aging. Phys. Rev. B.

[50] Zhang L.X., Ren X. (2005). In situ observation of reversible domain switching in aged Mn-doped BaTiO_3_ single crystals. Phys. Rev. B.

[51] Guo Y.Y., Yan Z.B., Zhang N., Cheng W.W., Liu J.M. (2012). Ferroelectric aging behaviors of BaTi_0.995_Mn_0.005_O_3_ ceramics: Grain size effects. Appl. Phys. A.

